# Incidence, predictors, and prognosis of premature discontinuation or switch of prasugrel or ticagrelor: the ATLANTIS - SWITCH study

**DOI:** 10.1038/s41598-019-44673-7

**Published:** 2019-06-03

**Authors:** Max-Paul Winter, Dirk von Lewinski, Markus Wallner, Florian Prüller, Ewald Kolesnik, Christian Hengstenberg, Jolanta M. Siller-Matula

**Affiliations:** 1 Department of Cardiology, Medical University of Vienna, Vienna, Austria; 2 Department of Cardiology, Medical University of Graz, Graz, Austria; 3 Lewis Katz School of Medicine, Temple University, Cardiovascular Research Center, Philadelphia, PA USA; 4 Clinical Institute of Medical and Chemical Laboratory Diagnostics, Medical University of Graz, Graz, Austria

**Keywords:** Cardiology, Outcomes research

## Abstract

Aim of the present study was to investigate the frequency and predictors of premature discontinuation or switch of ADP receptor blockers and its association with serious adverse events. For this purpose 571 consecutive ACS patients receiving ticagrelor (n = 258, 45%) or prasugrel (n = 313, 55%) undergoing PCI were enrolled in this prospective, observational, multicenter ATLANTIS-SWITCH substudy. Predictors of premature discontinuation or switch of antiplatelet therapy and their association with major adverse cardiovascular events and TIMI bleeding events were evaluated. Premature stop/switch was found in 72 (12.6%) patients: 34 (5.9%) stopped and 38 (6.7%) switched the ADP blocker. Ticagrelor treated patients were significantly more likely to stop/switch therapy as compared to prasugrel (15.9% vs. 9.2%, *p* = 0.016). We identified 4 independent predictors for stop/switch of ADP blocker: major surgery, need for oral anticoagulation (OAC), TIMI major bleeding and drug intolerance. TIMI major bleeding was a driver of stop/switch actions and occurred in 4.3% vs 0.2% in patients with vs without stop/switch (*p* = 0.001). The majority of stop/switch actions (75%) were physicians driven decisions. Importantly, stop/switch of therapy was not associated with increased risk of MACE (*p* = 0.936). In conclusion premature switch/stop of ADP blockers appears to be safe when mainly driven by physician’s decision and clinical indication.

## Introduction

Dual antiplatelet therapy (DAPT) of acetylsalicylic acid in combination with an adenosine diphosphate (ADP) inhibitor represents a significant advance in prevention of recurrent ischaemic events following drug eluting stent (DES) implantation^1–3^. Consequently, in patients with high ischemic risk like after acute coronary syndrome (ACS), current guidelines recommend DAPT for at least 12 month after DES implantation^4^. However, in real-world settings the adherence to recommended DAPT is uncertain and hard to assess. Some reports suggest that approximately 10% patients prematurely discontinue antiplatelet therapy within 30 days after stent implantation^5,6^. The reasons for premature discontinuation of DAPT within the first year after DES implantation of ACS are multifactorial. The therapy is either stopped by the patient or by a healthcare provider who may not realize benefits or the potentially severe consequences of antiplatelet therapy cessation^7,8^. However, especially in ACS patients non-adherence to medication may increase morbidity and mortality but also generates avoidable costs to the health care system^9^. Especially premature cessation of antiplatelet therapy has been associated with catastrophic ischemic events like stent thrombosis of recurrent ACS^10^. To date, there is only limited data on long-term adherence to DAPT therapy with novel antiplatelet agents in ACS patients. For this purpose we aimed to investigate the incidence and reasons of premature discontinuation or switch of antiplatelet therapy within 12 months after ACS in a multicenter, prospective registry of patients after acute myocardial infarction. Furthermore we investigated factors associated with premature discontinuation of ADP blocker or switch between agents and their association with adverse clinical events.

## Methods

### Study population and design

ATLANTIS - SWITCH substudy is a sub-investigation of the ATLANTIS-ACS (Association Between the Antiplatelet Drug Efficacy/Safety and Platelet Function in Patients Treated With Novel Platelet Inhibitors Due to an Acute Coronary Syndrome) study. In this prospective multicenter clinical study, it was planned to include 1000 consecutive ACS patients. Due to slow enrollment, the inclusion phase was terminated after inclusion of 571 patients from July 2012 until December 2016. The primary aim of the main study is to explore whether platelet reactivity in patients treated with potent platelet inhibitors is associated with clinical outcome; to investigate the incidence of adverse events under treatment with potent platelet inhibitors in the real life clinical scenario; and to investigate the association between genetic polymorphisms, inflammation, platelet reactivity and clinical outcome in long term follow-up.

Inclusion criteria were signed informed consent, ACS at admission, planned treatment with potent platelet inhibitors (ticagrelor and prasugrel), and age >18 years. The only exclusion criterion was participation in interventional trials. The study protocol complies with the Declaration of Helsinki and was approved by the Ethics Committee of the Medical University of Vienna and Medical University of Graz. Traditional cardiovascular risk factors were recorded according to the respective guidelines as previously described. All enrolled patients underwent a follow-up in person or by telephone contact 3, 6 and 12 months after study inclusion.

Most importantly the Austrian law stipulates all deaths to be recorded in the central Austrian death registry, which allows an almost complete follow up of all patients. Furthermore we performed a systematic exploration of the centralized patient management system of Vienna (AKIM- AKH-Informationsmanagement). This system offers a comprehensive chronological overview of patient data, documents, diagnoses and services as well as laboratory results acquired in Vienna general hospital as well as in every hospital of the Vienna hospital association (KAV). We systematically searched within this centralized platform for cardiovascular hospitalizations, re-catheterization, acute myocardial infarction, stroke, development of heart failure death. For this purpose we assume to present a virtually complete follow-up especially with regard to mortality^11^.

All patients received a loading dose of prasugrel (60 mg) or ticagrelor (180 mg) followed by a once daily dose of 10 mg prasugrel or two daily dose of ticagrelor (90 mg), respectively. The type of the ADP blocker used was at the discretion of the treating interventional cardiologist according to the current guidelines and the intern standard operating procedures (SOP) for the antiplatelet treatment of ACS patients in the both study centres. These SOP propose a following treatment algorithm: prasugrel was a drug of first choice in ST-elevation acute coronary syndrome patients (STE-ACS) or Non-ST-segment elevation acute coronary syndrome (NSTE-ACS) with diabetes, unless contraindicated, ticagrelor was a drug of first choice in the NSTE-ACS setting, unless contraindicated) as described previously^12–14^. Blood samples from patients were obtained at least one day after loading with prasugrel or ticagrelor at 8 a.m.

### Endpoints

The composite clinical endpoint of major adverse cardiovascular events (MACEs) included cardiovascular death, acute myocardial infarction, unstable angina, stent thrombosis, repeat revascularization and ischemic stroke. Myocardial infarction was defined based upon the 3rd Universal Definition of Myocardial Infarction^15^. Stent thrombosis was classified according to the Academic Research Consortium (ARC) criteria^16,17^. Unstable angina was defined as the presence of acute chest pain associated with ST depression, or new onset of negative T waves, and no elevation of troponins^18^. Ischemic stroke was defined as an episode of neurological dysfunction caused by focal cerebral, spinal, or retinal infarction with symptoms persisting ≥24 hours or until death^19^. All events were assessed via direct interview with the patients and verified using the centralized patients data management systems of the Vienna general hospital. Bleeding was classified according to the TIMI classification^20^ into four categories. TIMI I to III was pooled and used as the outcome parameter any bleeding. Drug adherence was assessed by review of the current medication and prescriptions of the patients.

#### Statistics

Risk factors, clinical data and categorical variables are presented as percentages of patients and were compared using χ2 or Fisher’s exact tests, as appropriate. Continuous data are expressed as median and interquartile range and compared using Mann-Whitney *U* test for two independent samples or Kruskal-Wallis test for more than two independent samples, with Bonferroni adjustment performed for multiple comparisons. The distribution of data was checked with the Kolmogorov-Smirnov test. The Kaplan-Meier (KM) method was utilized for construction of survival curves and to describe the incidence of switch or stop of antiplatelet therapy over one year or 60 days. The log-rank test was applied to evaluate differences between groups. Proportional Cox-regression analysis was used to adjust for confounding factors. Potential confounders (major bleeding, major surgery, oral anticoagulation, age, drug intolerance, smoking status, hypertension, STE-ACS, white blood cell count, fibrinogen levels and haemoglobin levels at admission) were entered into the Cox model on the basis of known clinical relevance or significant association observed at univariate analysis. Effect estimates were presented as hazard ratios (HR) and 95% CI. All tests were two-sided, a p-value < 0.05 was considered statistically significant. Calculations were performed using SPSS version 22.0 (IBM Corporation, Chicago, USA).

## Results

### Baseline characteristics

A total of 571 consecutive patients (Medical University of Vienna: 344 patients, Medical University of Graz: 227 patients) with acute myocardial infarction were enrolled in this multi-centre study (Table 1). Of those, seventy nine per cent were male, with a median age of 59 years (range: 51–69). Overall 258 (45%) received ticagrelor and 313 (55%) prasugrel as initial ADP-blocker. In this cohort, subjects receiving prasugrel as ADP-blocker (as compared to ticagrelor treated individuals) were significantly younger (57 years [IQR 50–66] vs. 63 years [IQR 54–73], *p* < 0.001), were significantly more often smokers (58% vs. 56%, *p* < 0.001) but significantly less often hypertensive (58% vs. 68%, p = 0.009). Within the study cohort 81.4% received a drug eluting stent, 10.2% a bare metal stent and 8.4% did not receive any stent.

**Table 1:** Baseline characteristics of the study cohort.

	Overall (n = 571)	Ticagrelor (n = 258)	Prasugrel (n = 313)	*P*-value*
**Baseline characteristics/Risk factors**				
Age, median years (IQR)	59 (51–69)	63 (54–73)	57 (50–66)	**<0.001**
Male gender, n (%)	450 (78.8)	184 (71.3)	266 (85.0)	0.674
BMI, kg/m^2^ (IQR)	27.5 (24.6–31.0)	27.5 (24.6–31.0)	27.5 (25.6–30.1)	0.232
Current smokers, *n* (%)	379 (66.4)	144 (55.8)	235 (57.8)	**<0.001**
Hypertension, n (%)	356 (62.3)	175 (67.8)	181 (57.8)	**0.009**
Diabetes II, n (%)	111 (19.4)	54 (20.9)	57 (18.2)	0.436
Dyslipidemia, n (%)	297 (52.0)	144 (55.8)	153 (48.9)	0.082
**Medication**				
Beta Blockers n (%)	488 (85.5)	218 (84.5)	270 (86.3)	0.366
ACE inhibitors n (%)	493 (86.3)	219 (84.9)	274 (87.5)	0.816
Statins n (%)	533 (93.3)	238 (92.2)	295 (94.2)	0.404
**CAD variables**				
STE-ACS, n (%)	369 (63.9)	87 (33.7)	282 (90.1)	**<0.001**
Previous AMI, n (%)	100 (17.5)	47 (18.2)	53 (16.9)	0.714
Multi vessel disease, n (%)	251 (44.0)	111 (43)	x	0.610
Family history of CAD, n (%)	204 (35.7)	82 (31.8)	122 (39.0)	0.065
**Procedural characteristics**				
Multivessel disease n (%)	251 (43.9)	111 (43.0)	140 (44.7)	0.882
No. of implanted stents (SD)	1.5 (1.0)	1.6 (1.1)	1.5 (0.9)	0.752
Total stent length mm (IQR)	27 (18–40)	26 (18–43)	28 (18–38)	0.861
Implanted DES n (%)	465 (81.4)	200 (77.5)	265 (88.4)	0.533
**Laboratory variables**				
TNT ng/L (IQR)	0.69 (0.05–57)	0.61 (0.07–58.00)	0.98 (0.05–54.62)	0.642
CRP mg/dL (IQR)	0.89 (0.27–5.80)	1.09 (0.27–8.01)	0.75 (0.27–4.80)	0.373
Creatinine mg/dL (IQR)	0.96 (0.83–1.20)	0.96 (0.82–1.25)	0.96 (0.85–1.16)	0.934
Fibrinogen mg/dL (IQR)	347 (278–424)	374 (295–442)	339 (217–410)	**0.026**
Hemoglobin mg/dL (IQR)	14.3 (12.9–15.2)	14.0 (12.2–15.0)	14.5 (13.4–15.6)	**<0.001**
White blood cell count g/L (IQR)	10.4 (8.4–13.0)	9.8 (7.5–12.6)	11.1 (9.2–13.6)	**<0.001**
Platelets g/L (IQR)	213 (171–255)	218 (171–256)	212 (171–254)	0.504

Continuous variables are given as medians and interquartile ranges (IQR). Counts are given as numbers and percentages, P-values are calculated using Mann- Whitney statistics. BMI body mass index, ACE angiotensin converting enzyme, STE-ACS ST elevation acute coronary syndrome, CAD coronary artery disease, DES drug eluting stent, TNT troponine T, CRP c reactive protein.

The majority of prasugrel treated patients presented with STE-ACS (90% vs. 34%, *p* < 0.001). There was no significant difference in cardiovascular pre-medication between patients that received ticagrelor or prasugrel at baseline. Of all investigated laboratory variables prasugrel treated patients displayed significantly higher levels of haemoglobin (14.5 mg/dL [IQR 13.4–15.6] vs. 14 mg/dL [IQR 12.2–15.0] and white blood cell count (11.1 g/L [IQR 9.2–13.6] vs. 9.8 g/L [IQR 7.5–12.6] but lower levels of fibrinogen (339 mg/dL [IQR 217–410] vs. 374 mg/dL [IQR 295–442].

### Switch or stop of ADP-blocker therapy during follow up

**Figure 1:**
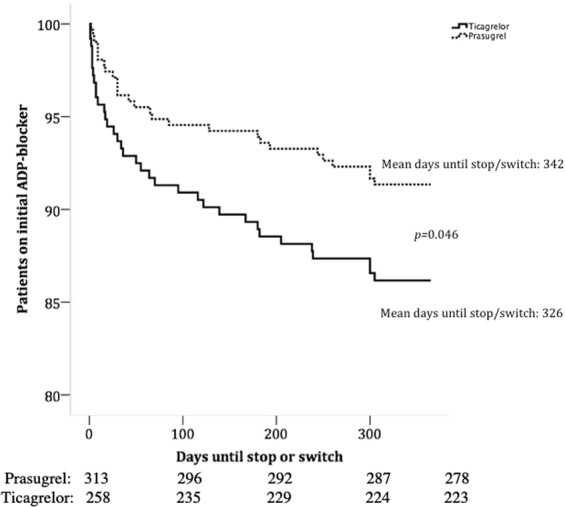
Kaplan Meier Time-to-Event curve for the occurrence of premature ADP-blocker discontinuation or switch.

Overall we found a satisfying patient adherence to ADP-blocker therapy with 501 (87.7%) of all patients taking index medication over the whole observational period. We found that patient initially treated with ticagrelor significantly more often switched or stopped the medication as compared to prasugrel treated patients (15.9% vs. 9.2%, *p* = 0.016). We could identify six different indications to prematurely discontinue ADP-blocker therapy (Table 2). Overall, there was no difference in the reasons to stop/switch the therapy between prasugrel and ticagrelor treated patients (*p* = 0.530) (Table 2). We observed in 65.7% of all cases a clear clinical indication to stop or switch ADP blocker therapy. Most importantly, there was no difference regarding the composite endpoint MACE or any TIMI bleeding event, in those who stopped/switched the therapy (Table 3). Kaplan Meier analysis revealed a mean adherence time was 16 days longer for prasugrel than for ticagrelor: 342 vs. 326 days, respectively (Log Rank *p* = 0.046) (Fig. 1). In those who stopped/switched the initial treatment, the mean time until stop or switch was 87 days for ticagrelor and 105 days for prasugrel (*p* = 0.502).

**Table 2:** Reason for premature discontinuation or switch of ADP-blocker therapy, Drug intolerance entails dyspnea, allergy, and rhythm disorder.

ADP Blocker	Reason to stop or switch ADP blocker						
	*P-*Value (overall)	Major surgery	Patients decision of unknown reason	Physician decision of unknown reason	OAC (+ASS)	Any bleeding	Drug intolerance
Ticagrelor n = 41 (15.9%)	0.530	4 (9.8)	10 (24.4)	5 (12.2)	10 (24.4)	4 (9.8)	8 (19.5)
Prasugrel n = 29 (9.2%)		1 (3.4)	12 (41.4)	2 (6.9)	7 (24.1)	4 (13.8)	3 (10.3)

Any bleeding entails TIMI minimal, minor, or major events. P > 0.05 for comparisons between prasugrel and ticagrelor groups.

**Table 3:** Bleeding and MACE incidence in patients that stopped or switched index medication as compared to those who stayed on the medication according to the primary used ADP blocker.

	Adherence to index medication n = 501 (88%)	Subjects with stop or switch n = 70 (12%)	*p-*value for comparison between those who stopped/switched vs. adherent to primary ADP blocker	Ticagrelor patients with stop or switch n = 41 (15.9)	Prasugrel patients with stop or switch n = 28 (9.2)	*p-*value for comparison between prasugrel and ticagrelor
Any bleeding n (%)	162 (32.3)	25 (35.7)	0.595	12 (29.3)	13 (44.8)	0.181
TIMI-major n (%)	**1 (0.2)**	**3 (4.3)**	**0.001**	2 (4.9)	1 (3.4)	0.771
TIMI-minor n (%)	5 (1.0)	0 (0)	0.404	0 (0)	0 (0)	
MACE	39 (7.8)	7 (10%)	0.523	4 (9.8)	3 (10.3)	0.936

### Predictors of premature ADP-blocker discontinuation

In the multivariate model, major surgery [HR: 36.37 (6.71–197.22), *p* < 0.001], need for oral anticoagulation [64.95 (20.28–207.99), *p* < 0.001], drug intolerance [HR: 56.18 (19.15–165.84), *p* < 0.001], and major bleeding [HR: 59.13 (9.68–361.04), *p* = 0.002] remained strong and independent predictors for premature ADP-blocker discontinuation (Table 4). Major bleeding led to 4.3% of stop/switches in the group who stopped/switched, whereas it occurred in 0.2% in those who did not stop/switch (*p* < 0.001) and was not as associated with the used ADP blocker (*p* = 0. 846) (Table 3).

**Table 4:** Predictors of premature ADP-blocker discontinuation. Any bleeding: TIMI minimal, minor and major.

Cox Regression	Premature stop of Switch/Stop of ADP-blocker therapy			
	Univariate		Multivariate	
	Crude HR (95% CI)	*P-*Value	Adj. HR (95% CI)	*P-*Value
Initial treatment strategy: ticagrelor vs prasugrel	0.60 (0.36–0.99)	**0.049**	1.720 (0.68–4.35)	0.252
Drug intolerance	29.817 (13.14–67.67)	**p** < **0.001**	56.18 (19.15–164.84)	**p** < **0.001**
Major surgery	8.32 (2.60–26.65)	**p** < **0.001**	**36.37 (6.71–197.22)**	**p** < **0.001**
Major bleeding on the initial P2Y12 blocker	9.64 (2.35–39.55)	**0.002**	**59.13 (9.68–361.04)**	**p** < **0.001**
Need for OAC	34.36 (18.30–64.50)	**p** < **0.001**	**64.95 (20.28–207.99)**	**p** < **0.001**
Age	1.02 (1.00–1.04)	**0.036**	1.016 (0.99–1.05)	0.283
Smoking	0.81 (0.48–1.36)	0.432	2.09 (0.93–4.68)	0.073
Hypertension	0.89 (0.53–1.48)	0.663	0.86 (0.45–1.65)	0.655
STE-ACS	1.49 (0.91–2.47)	0.116	2.55 (0.98–5.99)	0.54
White blood cell count	1.01 (1.00–1.01)	0.073	1.06 (0.99–1.01)	0.310
Fibrinogen levels mg/dL	1.00 (0.99–1.00)	0.265	1.00 (0.97–1.04)	0.854
Haemoglobin levels g/dL	1.01 (0.90–1.13)	0.840	1.07 (0.89–1.28)	0.479

### Premature stop of ADP-blocker therapy

**Figure 2:**
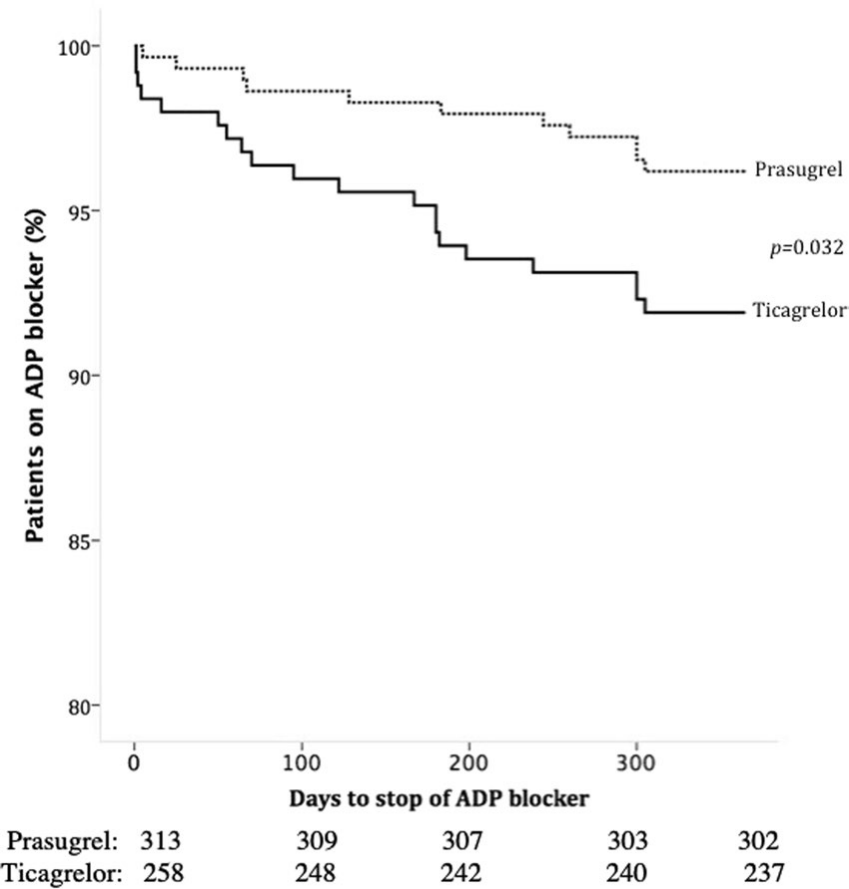
Kaplan Meier Time-to-Event curve for the occurrence of premature ADP -blocker stop.

During the follow up of one year, 34 (5.9%) patients prematurely stopped ADP-blocker therapy. Ticagrelor treated patients were significantly more likely to stop ADP-blocker therapy as compared to prasugrel treated patients (8.5% vs. 3.8%, *p* = 0.035). We could identify six different indications for drug discontinuation and there was overall no difference in reason to stop ADP-blocker therapy between ticagrelor and prasugrel study cohorts (Table 5). Kaplan Meier analysis revealed a mean time until discontinuation of ADP-blocker therapy of 337 days for ticagrelor and 352 for prasugrel treated patients (Log Rank *p* = 0.032) (Fig. 2).

**Table 5:** Reasons to stop ADP-blocker therapy, Drug intolerance entails dyspnea, allergy, and rhythm disorder.

ADP Blocker	Reason to stop blocker						
	*P-*Value (overall)	Major surgery	Patients decision of unknown reason	Physician decision	OAC (+ASS)	Bleeding	Drug intolerance
Ticagrelor n = 22 (8.8%)	0.092	3 (13.6)	2 (9.1)	6 (27.3)	6 (27.3)	2 (9.1)	3 (13.6)
Prasugrel n = 12 (3.8%)		0 (0)	6 (50)	2 (16.7)	1 (8.3)	2 (16.7)	1 (8.3)

### Switch of ADP-blocker therapy during follow up

During the post-hospital follow-up period, 38 (6.7%) patients were switched from ticagrelor or prasugrel to clopidogrel (all were switched down to clopidogrel with no switches between prasugrel and ticagrelor). In contrast to premature stop, there was no significant difference in probability to switch to clopidogrel between ticagrelor and prasugrel treated patients (8.5% vs. 5.1%, *p* = 0.106), respectively.

Likewise we could identify six different indications for switching ADP-blocker therapy, but there was no significant difference between our two treatment cohorts (*p* = 0. 697) (Table 6).

**Table 6:** Reasons to switch ADP-blocker therapy, Drug intolerance entails dyspnea, allergy, and rhythm disorder.

ADP Blocker	Reason to switch ADP blocker						
	*P-*Value (overall)	Major surgery	Patients decision of unknown reason	Physician decision	OAC (+ASS)	Bleeding	Drug intolerance
Ticagrelor n = 22 (8.8%)	0.697	1 (4.5)	0 (0)	9 (40.9)	5 (22.7)	2 (9.1)	5 (22.7)
Prasugrel n = 16 (5.1%)		0 (0)	0 (0)	6 (37.5)	6 (37.5)	2 (12.5)	2 (12.5)

**Figure 3:**
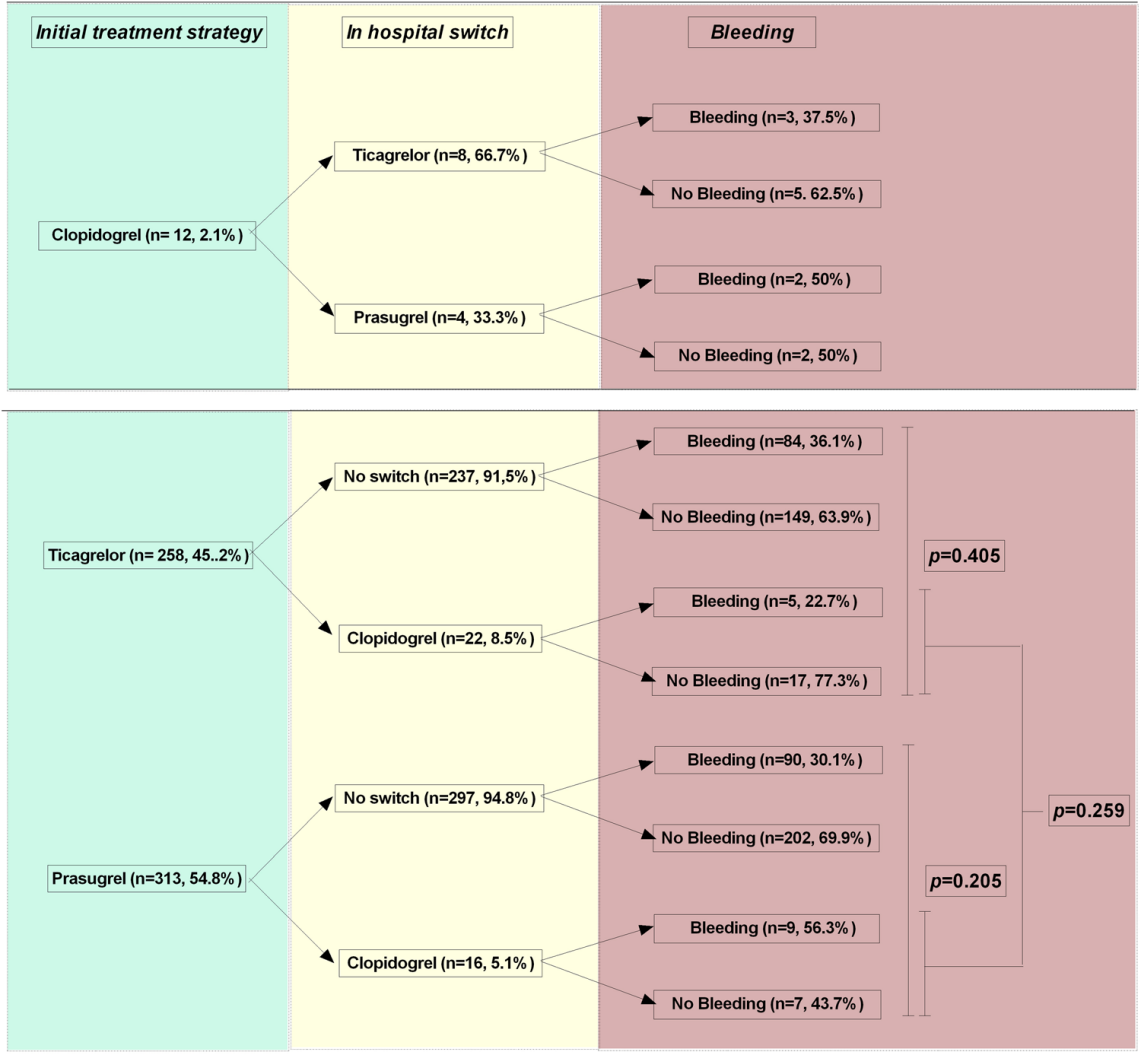
Switching regime according to initial ADP-blocker and subsequent bleeding events.

**Figure 4:**
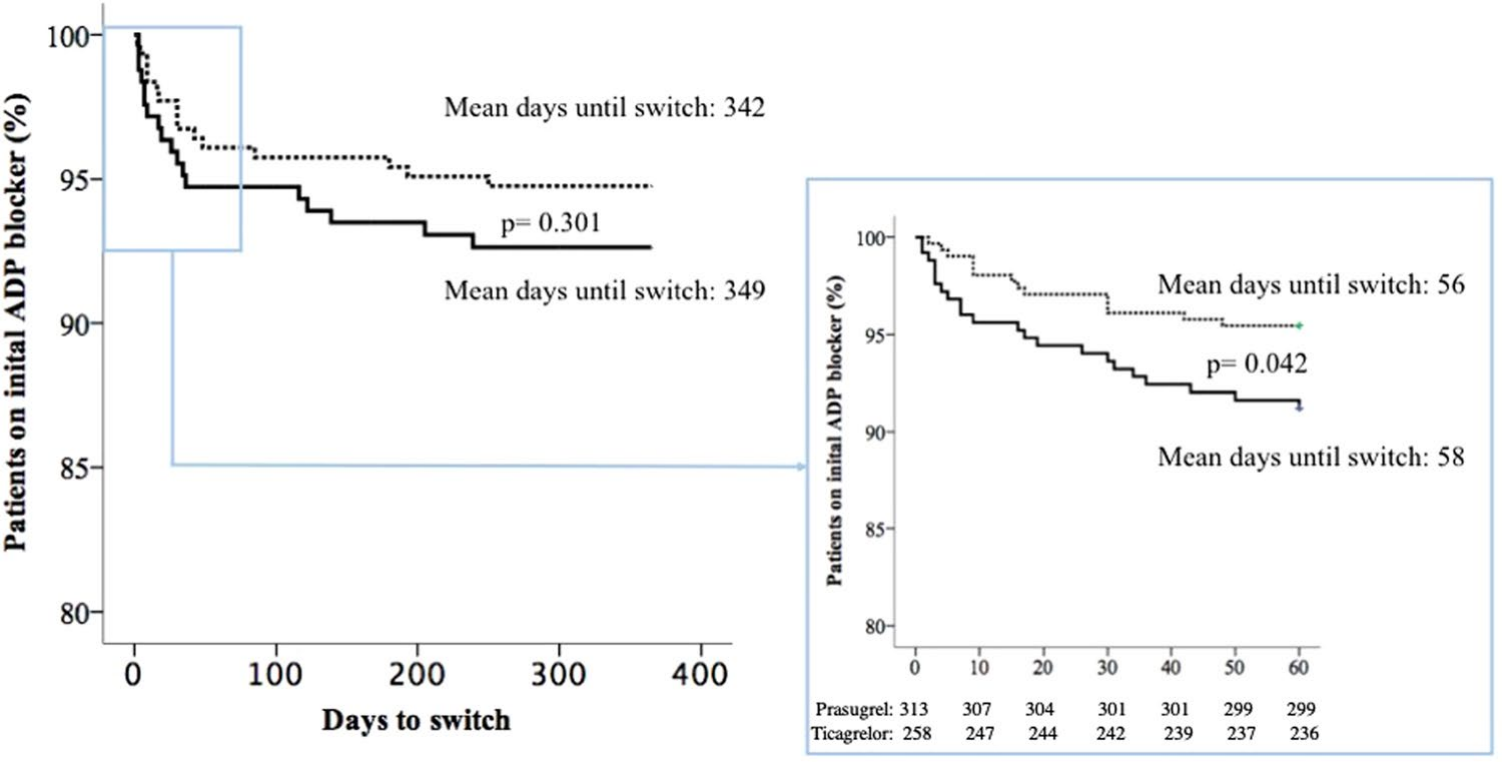
Kaplan Meier Time-to-Event curve for the occurrence of ADP-blocker switch for the whole observational period (A) and the initial phase of 60 days (B).

Moreover, after switch, there was no significant different rates for any bleeding in patients that were switched to clopidogrel, as compared to those who were not or those that were switched from clopidogrel to a novel ADP blocker (2.1%). Overall only 22 ticagrelor and 16 prasugrel treated patients were switched to clopidogrel, with five previously ticagrelor and nine previously prasugrel treated patients experiencing a bleeding event (Fig. 3A,B). Kaplan Meier analysis revealed no difference between ticagrelor and prasugrel treated patients in time to switch to clopidogrel of 342 days for ticagrelor and 349 for prasugrel treated patients (Log Rank *p* = 0.301) (Fig. 4A). As majority of all switches are prone to be in the initial phase of ADP-blocker treatment, we further investigated this vulnerable phase until day 60. In this time frame ticagrelor patients were more likely to switch to clopidogrel (8.5%) as compared to prasugrel (4.5%), with a shorter time to switch in the ticagrelor group *(p* = 0.042; (Fig. 4B).

## Discussion

The ATLANTIS - SWITCH substudy investigated the incidence and predictors of premature discontinuation of DAPT in 571 patients after ACS treated with novel platelet inhibitors. The main finding is that adherence to prescribed antiplatelet therapy after ACS is satisfactory and switch or stop of the treatment is mainly driven by physician’s decision and clinical indication.

After ACS with or without coronary stent implantation, 12 month of DAPT with potent platelet inhibitors instead of clopidogrel is recommended to reduce the risk of stent thrombosis or recurrent ischemic events^21,22^. The indication for a 12 month DAPT treatment are based on well conducted randomized clinical trials with high adherence to treatment as a consequence of proper patient selection, pre-scheduled follow-up visits and of course free provision of the investigated platelet inhibitors^1^. In clinical routine adherence to ADP blockers seems to be much lower. For instance, registries reported cessation rates for clopidogrel from 14% within the first month to 38% by 12 month^5,23–25^. The major adverse events associated with premature discontinuation of DAPT are stent thrombosis and recurrent ischemic events. The prognostic implications of these events are noteworthy and therefore identification of patients at risk for premature discontinuation and precautionary measure to prevent unnecessary cessation is crucial. In contrast to previous publications, we found that in two Austrian university hospitals only 5.9% of all patients prematurely stopped and 6.7% of them switched antiplatelet therapy within the first 12 month after ACS. Patients treated with ticagrelor as compared to prasugrel were more likely to stop ADP-blocker therapy (8.5% vs. 3.8%). Additionally ticagrelor treated patients revealed a significantly shorter mean adherence time as compared to prasugrel treated patients. In our cohort we found a “vulnerable phase” of sixty days within which ticagrelor patients were more likely to switch as compared to prasugrel. Both ticagrelor and prasugrel treated patients were more likely to switch as a consequence of TIMI major bleeding events.

Our findings go in line with observations of Rossini *et al*. reporting that early discontinuation of DAPT was predictable by in-hospital major bleeding in a cohort of 1,358 consecutive patients after DES implantation^24^. The combination of switch or stop of antiplatelet therapy as endpoint, revealed that ticagrelor treated patients were significantly more likely to prematurely stop/switch therapy as compared to prasugrel treated patients (15.9% vs. 9.2%), resulting in an average of 16 days longer treatment adherence for prasugrel than for ticagrelor. The reasons to prematurely switch or stop ADP blocker therapy are multifactorial. In line with previous reports we could identify six different indications for stop or switch of ADP blocker therapy^26^. In the majority of patients (24%), the ADP blocker therapy was stopped/switched due to the additional indication for oral anticoagulation. In recent studies non-reimbursement of the medication was a driver to stop ADP blocker therapy^25,26^. The Austrian law stipulates that all citizens receive publicly funded care, which allows an almost complete re-imbursement of prescribed treatments, leaving financial reasons to stop the therapy negligable. For this purpose, the majority of stop/switch actions (75%) were decision of the treating physician which is in line with previous reports that stated that in up to 90% the treating physician decision was the dominant reason reported^26^. Beside the indication for oral anticoagulation we could not identify specific reasons of the treating physician to stop or switch the antiplatelet agents. We assume that the increased risk of bleeding, observed in patients treated with novel ADP blockers is the most important consideration of the treating physicians when stopping the therapy. However this strategy is only reasonable in patients, where mortality risk due to bleeding outweighs the mortality risk due to recurrent major adverse cardiac events^26,27^. It is well evidenced that premature discontinuation of DAPT is associated with exacerbated risk for MACE^24,28^, stent thrombosis^29^, unplanned repeat revascularization^30^ and cardiovascular death^24^. In the present analysis we could observe that, stop/switch of antiplatelet therapy was not associated with increased risk of MACE (*p* = 0.936). We assume that this contradictory result is a consequence of the indication for stop or switch. Previous reports have shown that if ADP blocker discontinuation was indicated by the health care provider, it is not associated with increased risk of MACE^25^. As the majority of our patients had the reason to stop or switch of the ADP blocker and this was a decision of the treating physician, indicating in our cohort careful evaluation of the risk/benefit ratio resulting in a favourable outcome in these patients^31^. This finding is remarkable as it adds another safety aspect to the ongoing discussion on de-escalation of DAPT, which is defined as a switch from a potent antiplatelet agent (ticagrelor or prasugrel) to clopidogrel to prevent bleeding events. Overall it is known that greatest benefits of potent P2Y_12_ blockade occur early through reduction of ischemic risk and the risk of bleeding from these potent medications occur in the “maintenance phase of treatment”. Therefore, a stage-adapted strategy with the early use of potent P2Y_12_ blockade in acute treatment, followed by switching to clopidogrel during the maintenance phase seems appropriate. So far there are two large scale outcome studies namely the TOPIC (timing of platelet inhibition after acute coronary syndrome) randomized study and the TROPICAL- ACS study^32,33^. Both trials tested this approach and found that de-escalation do not result in increased risk for ischemic events as compared to DAPT with potent P2Y12 inhibitors. We could observe similar effects in our patients that were deescalated to clopidogrel, encouraging the strategy to switch the antiplatelet therapy in reflection of individual patient risk factors for bleeding or ischemic events.

## Conclusion

Major bleeding events, indication for anticoagulation, drug intolerance and major surgery were drivers of therapy cessation or switch in ticagrelor or prasugrel treated patients. Premature switch/stop of ADP blockers seems to be safe when mainly driven by physician’s decision and clinical indication.

### Limitations

The presents study harbors some important limitations. First, despite the relatively high number of prospectively enrolled patients, we only observed a small number of major adverse cardiac events. For this purpose the study might be underpowered for detecting differences in this endpoint and the relative findings need to be confirmed in even larger cohorts. Second, potential unmeasured confounders cannot be excluded. The exact downgrading strategy (switching from ticagrelor and prasugrel to clopidogrel) as a consequence of a treating physician decision is hard to assess. Finally, all patients in our study had been treated at high-volume tertiary care centres, and therefore the generalization of study results to other Countries and centres is unknown.
